# Investigating Electrical Impedance Spectroscopy for Estimating Blood Flow-Induced Variations in Human Forearm

**DOI:** 10.3390/s20185333

**Published:** 2020-09-17

**Authors:** Gautam Anand, Andrew Lowe

**Affiliations:** 1 Institute of Biomedical Technologies, Auckland University of Technology, Auckland 1010, New Zealand; andrew.lowe@aut.ac.nz; 2 School of Engineering, Computer and Mathematical Sciences, Auckland University of Technology, Auckland 1010, New Zealand

**Keywords:** electrical impedance spectroscopy, hemodynamic monitoring, artery diameter, ultrasound

## Abstract

This work aims to investigate the feasibility of employing multi-frequency bioimpedance analysis for hemodynamic assessment. Towards this, we aim to explore one of its implementations, electrical impedance spectroscopy (EIS), for estimating changes in radial artery diameter due to blood flow. Following from our previous investigations, here, we use a commercial device—the Quadra^®^ Impedance Spectroscopy device—for impedance measurements of the forearm of three subjects under normal conditions and occluding the artery with a cuff. This was performed simultaneously with ultrasound measurements as a reference. The impedance spectra were measured over time, yielding waveforms reflecting changes due to blood flow. Contributions from the fat/muscle domains were accounted for using the occluded impedance response, resulting in arterial impedance. A modified relationship was approximated to calculate the diameter from the arterial impedance, which showed a similarity with ultrasound measurements. Comparison with the ultrasound measurements revealed differences in phase and amplitude, primarily due to the approximated relationship between impedance and diameter and neglecting the impedance phase analysis. This work shows the potential of EIS, with improvements, towards estimating blood flow-induced variation in arteries. Further analysis and improvements could help place this technology in mainstream clinical practice for hemodynamic monitoring.

## 1. Introduction

Bioimpedance analysis (BIA) is an emerging noninvasive technology which uses the electrical properties of tissues to determine several physiological modalities. It is the gold standard for body fat/water composition and tissue/organ imaging, and presents itself as a candidate for noninvasive hemodynamic monitoring. Hemodynamic assessment involves measuring cardiac function through several parameters such as heart rate (HR), blood pressure (BP), stroke volume (SV) and cardiac output (CO). Hemodynamic monitoring primarily relies on the current gold standard—thermodilution (using pulmonary artery catheter (PAC)) [1,2,3,4]—which is an invasive method along with semi- or minimally invasive techniques employing Doppler echocardiography [5,6,7,8,9]. Due to concerns around patient comfort and continuous measurements, alternatives to the existing hemodynamic assessment methods have always been of interest [10,11]. BIA offers one such truly noninvasive, low-cost implementation method; however, it has not been able to establish itself as a reliable assessor.

From a measurement perspective, BIA is implemented as either single-frequency BIA (SFBIA) or multifrequency BIA (MFBIA). SFBIA implementations include applications such as impedance cardiography (ICG), which is currently used for hemodynamic monitoring [12,13]. MFBIA includes applications such as electrical impedance tomography (EIT), an imaging technique [14,15,16] to detect, for example, breast cancer; and electrical impedance spectroscopy (EIS), for fat/water composition analysis [17,18]. ICG involves the application of a low alternating excitation current at a frequency to the thorax, and the response is used to monitor hemodynamics through measured changes in impedance. It requires simultaneous measurement of the electrocardiogram (ECG) signal to calculate SV, HR and CO. This technique has its advantages as a noninvasive, easy-to-operate system and is hence widely marketed as an effective tool for monitoring hypertension and heart failure. However, it does not allow an accurate reconstruction of the complete blood profile, which, in many instances, carries important information, especially for continuous monitoring. Mixed results have been reported for the reliability of ICG on comparison with the existing gold standard for hemodynamic monitoring [19,20,21,22,23,24,25,26]. This is largely due to the original implementation of ICG by Kubicek et al. [27,28], an analysis which was based on many assumptions [29,30]—cylindrical modelling of the thorax region and neglecting the contributions of the adjacent tissue layers such as fat and muscle.

## 2. Prior Work

**Figure 1:**
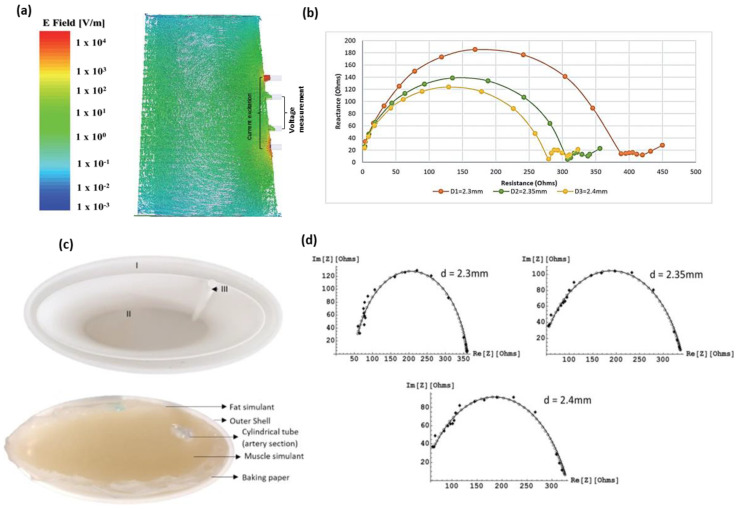
Summary of the results from our preliminary studies. (**a**) Electric field distribution from the simulation study at 50 kHz. (**b**) Simulation-obtained Cole plots for impedance spectra at three different diameters. (**c**) Preparation of the forearm phantom (I—Fat, II—Muscle and III—Artery). (**d**) Comparison of the simulation and phantom results with the same material properties.

Towards establishing BIA as a reliable tool of hemodynamic monitoring, our research aims to investigate the potential of MFBIA techniques such as EIS for estimating blood flow-induced physiological variations. Here, we follow from our preliminary investigations—a simulation study and a human forearm phantom study. To explore the potential of MFBIA, we initially followed a simulation approach for modelling the impedance response of a human forearm section with different diameters of the radial artery [31]. The simulation study consisted of modelling three layers of the forearm—fat, muscle and blood. The objective was to understand the distribution of electric fields within the tissue domains in the forearm cross-section and obtain the impedance spectra for three different arterial diameters, 2.3, 2.35 and 2.4 mm. The simulation was performed using the Ansys Electromagnetics suite (R2016) to excite the geometry between 1 kHz and 2 MHz with a current of 1 mA. Figure 1a shows the electric field distribution obtained for the geometry at 50 kHz along with impedance results at all three arterial diameters shown in Figure 1b. A clear distinction was observed between the impedance spectra of the three diameter instances. The very-low-frequency responses were not found to follow the Cole plot trend; however, it was experienced for most of the frequency range. The different values of arterial diameter mimicked blood flow-induced changes to an extent where the volumetric changes due to blood dynamics were reflected in the impedance spectra.

The simulation investigation was followed by an experimental simulation on a human forearm phantom [32]. The forearm phantom was developed—constituting the same three layers as in the simulation study: Fat, muscle and blood—and impedance measurements were taken with a provision for the same three arterial diameters. The tissue simulants were realized through easily available ingredients like gelatine, agar, NaCl, deionized water and propylene glycol. The measurements were performed using a commercially available Quadra^®^ Impedance spectroscopy device between 1 and 349 kHz, and eventually extrapolated to 2 MHz. Figure 1c shows the phantom preparation setup, and the final results are shown in Figure 1d. Although the initial results showed similar behaviour to the simulation study (higher magnitude of the impedance spectra at lower arterial diameter), they deviated significantly from the values obtained through the simulation analysis because of differences in the dielectric dispersion of developed tissue simulants from those modelled in the simulation, as well as electrode size. However, excellent agreement was obtained once the simulation was revised with the same properties as the developed simulants.

Both these works identified the potential for employing MFBIA in hemodynamics. Although the artery under consideration (radial artery) is smaller than many other arteries, the ease of measurement makes it feasible to be investigated in any setting. All these factors motivated us to proceed to the present investigation of analysing the spectral changes of the forearm tissues in real-time and quantify the response of blood in terms of changes in arterial dimensions.

This work builds on our findings from the above-mentioned research towards experimentation on an actual human forearm. The objective of this work is to evaluate a new EIS sensing approach for estimating changes in the left radial artery due to pulsatile blood flow. These measurements will be compared to the ultrasound-derived changes in the arterial diameter. While this work is not a complete validation of the procedure, it is intended to present a new technological approach towards an accurate estimation of blood flow-induced impedance variations, and hence, reliable markers for hemodynamic monitoring.

## 3. Materials and Methods

**Figure 2:**
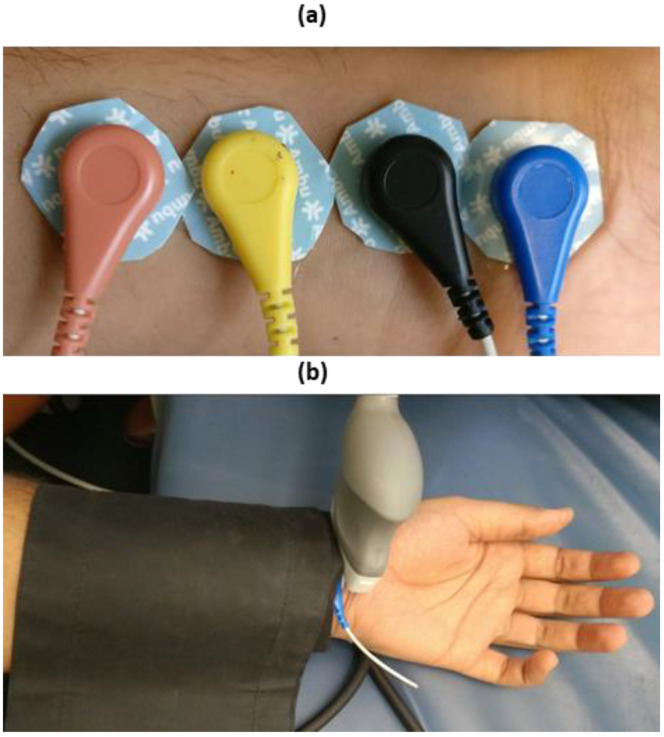
(**a**) Electrode setup for impedance measurements at forearm section. (**b**) Ultrasound and impedance measurement setup with cuff pressure at 200 mmHg.

For the purpose of investigation, the study was performed on three subjects—all healthy males (aged 26, 42 and 67) under normal resting conditions, which were members of the research team. After consultation with the institutional ethics committee, the scope of this investigation was deemed exempt from ethics approval. The experimental procedure was set-up to measure the impedance response of the ventral side of the forearm section within a 10 cm span from the wrist. The placement of electrodes was chosen to mimic the configuration used in the simulation study. The separation was 1 cm between each electrode, where the outer pair was used to feed the input current and the inner pair to measure the voltage, as can be seen in Figure 2a.

The electrode setup was connected to the Quadra^®^ impedance spectroscopy device under a similar configuration to that used for the phantom experiments. The frequency range of measurements was 1–349 kHz. The impedance measurements were taken under conditions of normal blood flow and artery occlusion. The artery was occluded by using a sphygmomanometer (Accoson^TM^ MK2 BS 2744, Accoson, UK) with a maximum cuff pressure of 200 mmHg. The cuff was placed over the electrodes. Two sets of measurements were performed, with occlusion and without occlusion. The measurements without occlusion were performed to reflect the total impedance response of the blood flow in the radial artery and the fat and muscle tissues surrounding it.

Artery occlusion was performed to squeeze the blood out of the artery at the measurement site and reflect the impedance of only the fat/muscle tissues. This was intended to acquire two sets of measurements to separate the contribution of blood flow from the overall impedance response.

The impedance spectra obtained using the Quadra^®^ impedance spectroscopy device was collected and analysed using MATLAB. The diameter for the left radial artery was measured for all the three subjects by ultrasound (Acuson Sequoia^TM^ C512, Siemens Healthcare, Erlangen, Germany) using a 17L5 HD probe. The high-resolution probe (17L5) was employed with frequency capabilities up to 15 MHz and was suitable for obtaining high-resolution M-mode images at the superficial depths (up to 2 cm). The probe was placed transverse to the arterial section to acquire cross-sectional images, and the diameter changed with blood flow. The ultrasound measurements were performed under the condition of no arterial occlusion by the cuff along with the impedance measurements, as can be seen in Figure 2b.

### Mathematical Analysis

To separate the impedance contribution of blood flow in the radial artery from the combined impedance measurement of the forearm (impedance without artery occlusion), a parallel combination of the impedance of the artery and the remaining tissue layers of fat and muscle was assumed (similar to that assumed by Webster [33]). The applicability of this model (with separate fat and muscle domains) was separately analysed [34] and supported by the obtained results. This was based on the consideration of two factors—complex conduction pathways through the considered tissue domains and the ability to isolate impedance variations due to blood flow in the artery. The arterial domain is more conductive than either muscle or fat (allowing more sensitivity to pulsatile blood flow-induced changes) and better defined in geometry. Therefore, considering the possibility of an artery extending through both muscle and fat, as well as the varying degree of perfusion of fat and muscle tissues, the impedance of the pulsating radial artery was taken to be $Z_{a}$ and that of the fat/muscle layers to be $Z_{f/m}$, leading to:

(1) $$\frac{1}{Z_{t}}=\frac{1}{Z_{a}}+\frac{1}{Z_{f/m}}$$

where $Z_{t}$ is the total impedance of the forearm. Under normal conditions, i.e., measurement without the cuff, the total impedance will be $Z_{t}$, whereas, with the artery occluded, the impedance will not include contributions from blood flow and will, hence, only be $Z_{a}$. Therefore, the impedance of the artery can be calculated as:

(2) $$Z_{a}=\frac{1}{\frac{1}{Z_{t}}-\frac{1}{Z_{f/m}}}$$

From the above measurement procedure, the impedance measurement without the cuff was considered to be $Z_{t}$ and the measurement with the artery occluded was considered as $Z_{f/m}$.

Moreover, considering the radial artery to be cylindrical in the section leads to a relation between the impedance magnitude and the arterial diameter, stated as:

(3) Za=1σ∗lA= 1σ∗ lπd24

where σ is the conductivity of blood (0.7 S m^−1^), l is the length of the section of the artery under consideration and A is the cross-sectional area of the radial artery (A=πd24, where d is the arterial diameter). In this case, l = 0.01 m; hence, the diameter of the artery can be calculated as:

(4) $$d\;\left(in\;\mathrm{mm}\right)=135*\sqrt{\frac{1}{Z_{a}}}$$

To relate the calculated diameter values more closely to the actual diameter of the radial artery, (4) was modified to:

(5) $$d\left(in\;\mathrm{mm}\right)=k*135*\sqrt{\frac{1}{Z_{a}}}+c$$

where *k* is the proportionality constant and *c* is the offset. This linear scaling is thought to compensate for two aspects. Firstly, when the cuff is inflated, blood is removed not only from the artery under consideration but from the venous system and other perfused tissue. Therefore, the impedance being measured using the method above represents both the volumetric changes due to blood flow and a component of nonpulsatile blood (from veins and other perfused tissues). Secondly, as observed from the distribution of the electric field inside the tissue domains during the simulation study, the conduction path was identified to be normal to the forearm fat and muscle layers before and after traversing through the arterial section, which cannot be exactly represented as a parallel combination of tissue domains. These two unmodelled considerations do not change with time and are taken into account by considering the above-mentioned correction factors (*k* and *c*), although it is recognised that more investigation into these effects is warranted.

On calibrating the average value of the calculated diameter and calculated maximum change in diameter during flow (for each subject) with the expected average value (2.3 mm) and average changes in it (0.5 mm) from the literature, respectively, the values were approximated to be 0.15 for *k* and 1.5 for *c*. Hence, the modified relation of arterial diameter to the impedance was taken to be:

(6) $$d\left(in\;\mathrm{mm}\right)=20.25*\sqrt{\frac{1}{Z_{a}}}+1.5$$

## 4. Results

### 4.1. Impedance Measurement Results

**Figure 3:**
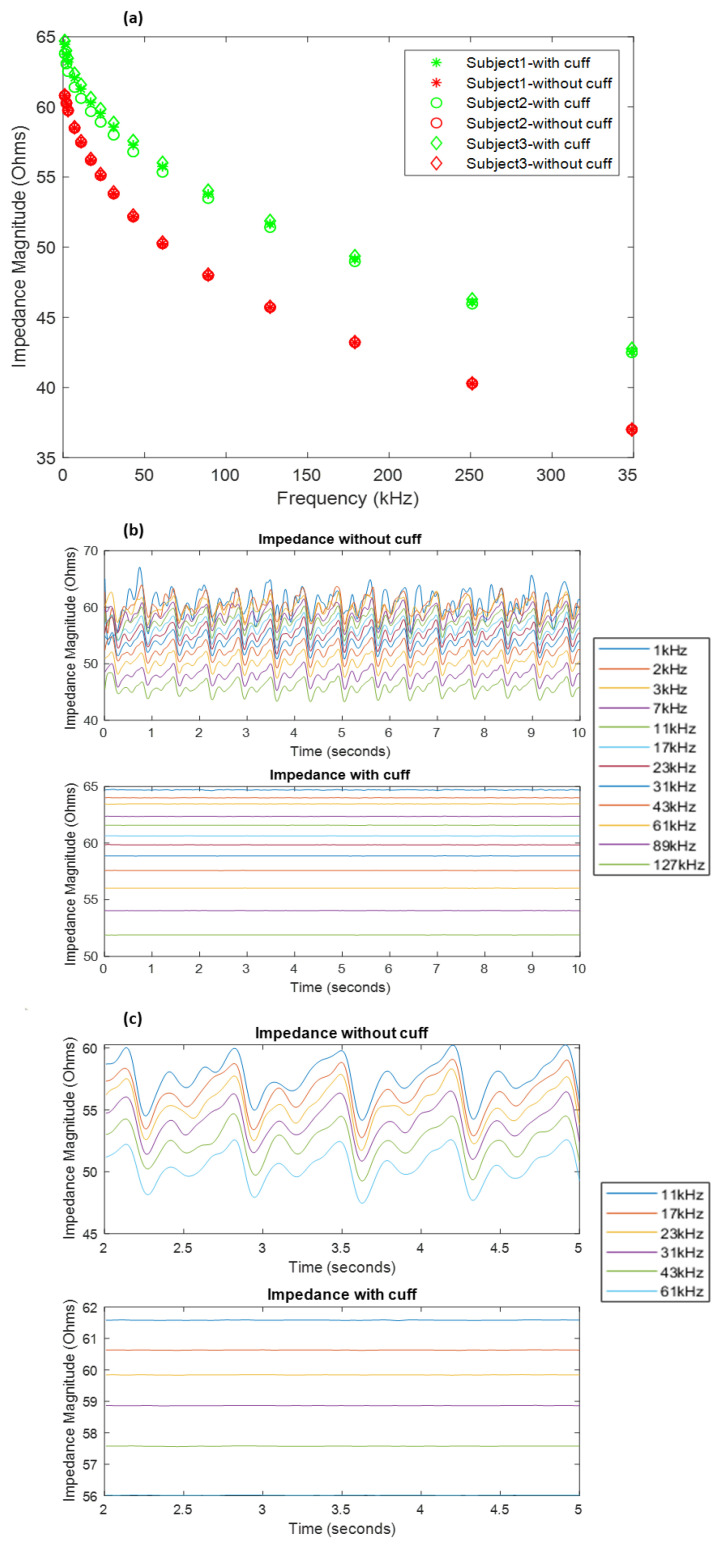
(**a**) Impedance spectra for subjects 1, 2 and 3 as measured by Quadra^®^ impedance spectroscopy device, with and without the application of cuff pressure. (**b**) Impedance waveforms measured within 1–127 kHz for subject 3, without and with cuff application. (**c**) Detailed insight into impedance waveforms within 11–61 kHz within a time interval of 2 to 5 s.

The measurements obtained under normal conditions and the artery occluded with the cuff pressure at 200 mmHg have been shown in Figure 3a. It shows a significant difference in the impedance of the forearm under the two conditions. The overall magnitude of impedance was found to be larger in the case of the occluded artery (shown in green) than the normal conditions (shown in red). This is expected due to the relatively low impedance of blood compared to other tissue.

The Quadra^®^ impedance spectroscopy device measured the impedance spectra at a rate of 1000 samples/s. This facilitated the time domain analysis of the impedance values. The impedance spectra samples for each subject were recorded for 10 s, which were analysed for each frequency. The collected samples were processed to extract the impedance contributions due to blood flow by passing time series through a low-pass filter with a cut-off of 7.5 Hz followed by high-pass filtering above 0.7 Hz to remove baseline wander. An example of the resultant signals obtained has been shown in Figure 3b for subject 3. Figure 3c shows a more detailed insight into some of the waveforms at a few frequencies.

**Figure 4:**
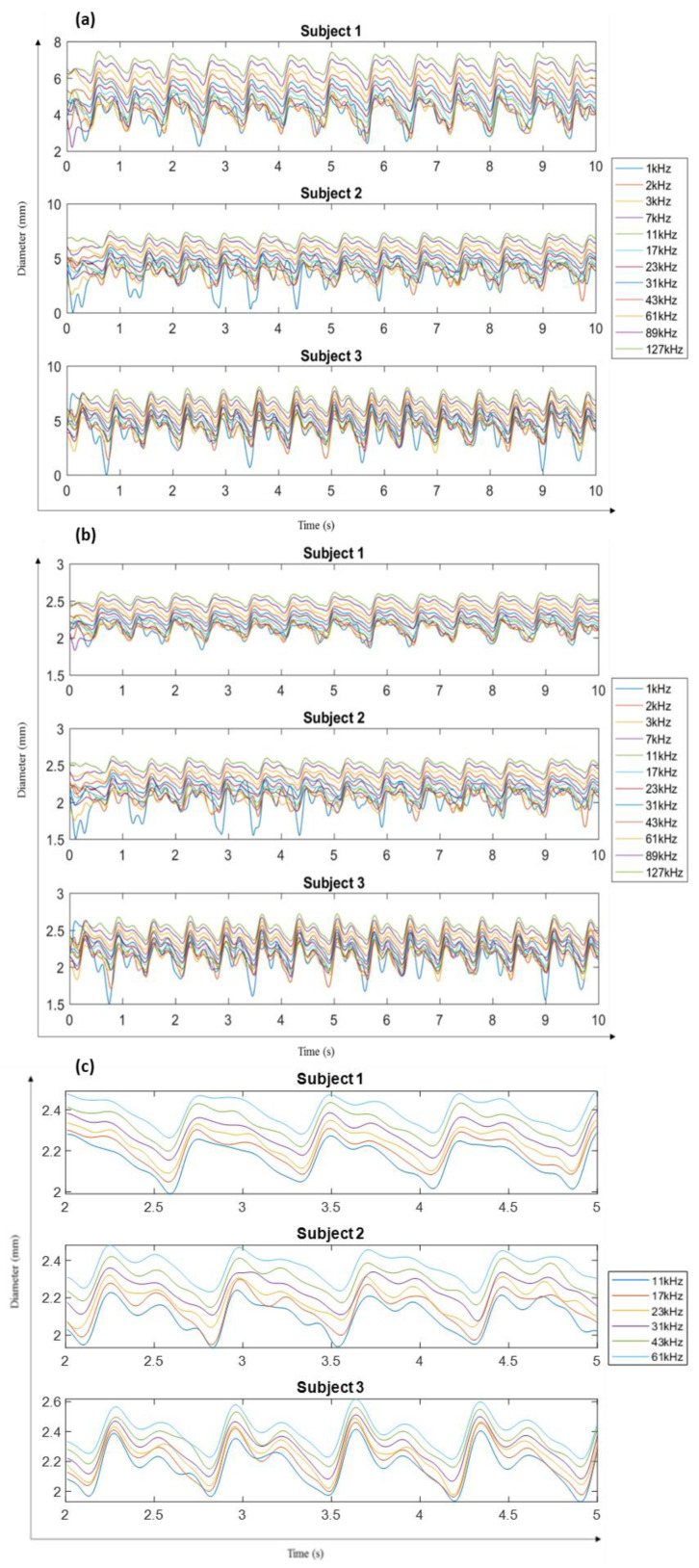
(**a**) Diameter waveforms calculated using impedance from (4) for each subject. (**b**) Modified diameter waveforms calculated using (6) for each subject. (**c**) 3 s section of modified diameter waveform calculated for each subject for frequencies within 11–61 kHz.

From the obtained impedance signals, the application of cuff pressure was reflected in negligible variations in signal amplitudes as compared to quite significant and periodic waves in the other case. The acquired impedance waveforms can be seen to be noisy at lower frequencies of 1–3 kHz. In addition, the impedance for the frequencies 179, 251 and 349 kHz was too noisy to be considered. Hence, only the signals up to 127 kHz were considered for further analysis. Following the expression in (4), the obtained diameter waveforms for each subject can be seen in Figure 4a. The final diameter waveforms of the radial artery for all three subjects within the frequencies of 1–127 kHz were obtained using (6), as seen in Figure 4b. In addition, Figure 4c shows the diameter waveform for all subjects between the 2nd and 5th second to indicate more clearly the morphology of the diameter changes due to blood flow as obtained from the measured impedance.

### 4.2. Ultrasound Measurement Results

**Figure 5:**
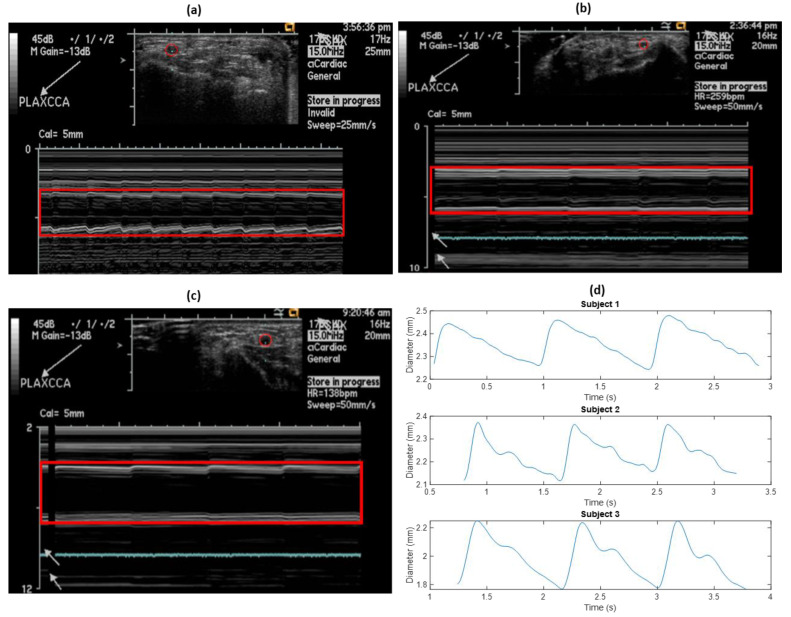
(**a**–**c**) Radial artery diameter changes measured using ultrasound for subjects 1, 2 and 3, respectively. The artery is outlined by a red circle and the corresponding M-mode diameter wave is outlined using a red rectangle. (**d**) Extracted diameter waveforms for the subjects from ultrasound measurements.

As a reference, ultrasound measurements to estimate the diameter of the left radial artery were performed on all three subjects, at the same time as the impedance measurements. Initially, the probe was adjusted to correctly locate the cross-section of the artery for M-mode analysis. Figure 5a–c show the cross-sectional diameter changes in the left radial artery for all the three subjects, as captured from the ultrasound.

Extraction of the diameter waveform was performed using image processing in MATLAB. Each ultrasound image was smoothened, and contours were extracted containing the information of each related pixel. The contours for the near- and far-artery walls (on the probe) were identified, and the overall diameter waveform was obtained as a difference between the two. The extracted diameter waveform from the above ultrasound images for all the subjects can be seen in Figure 5b.

## 5. Discussion

The diameter calculated from the impedance measurements was different for different frequencies but possessed a similar waveform morphology to that measured by ultrasound, thus highlighting the potential of impedance measurements for estimating arterial diameter changes.

### 5.1. Comparing Ultrasound Measurements with Simulation Results

**Figure 6:**
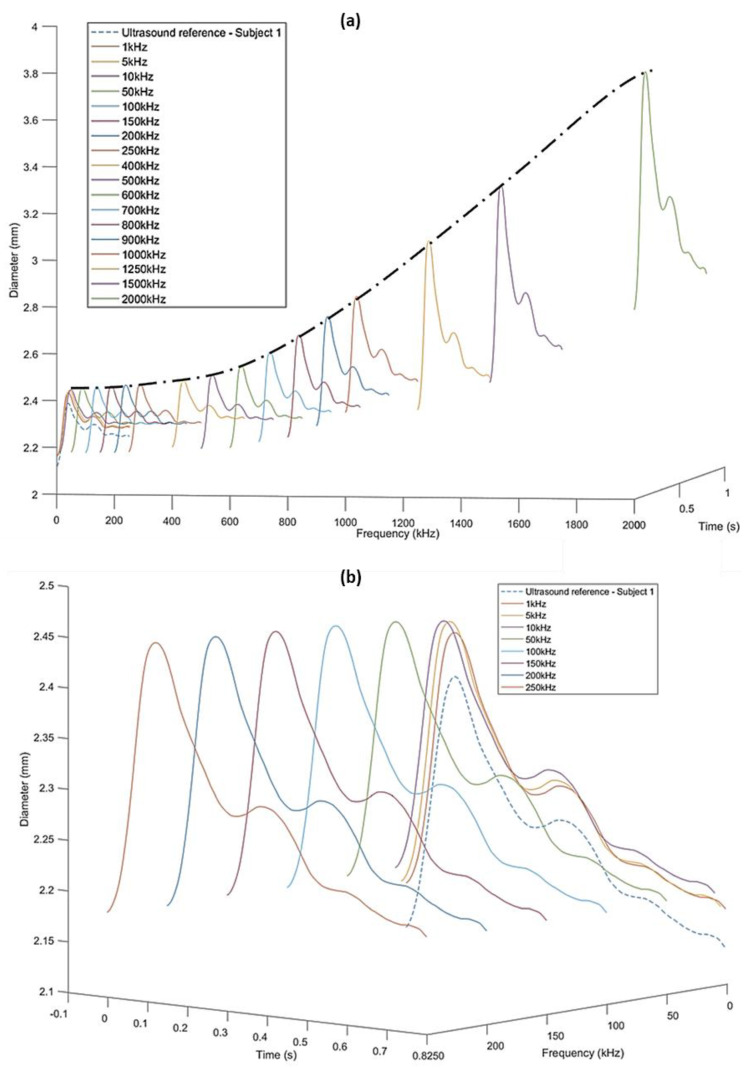
(**a**) Increasing trend of diameters obtained using simulation response for frequencies 1 kHz–2 MHz compared to the ultrasound-measured diameter for subject 1 (dashed line). (**b**) Simulation-obtained diameters within 1–250 kHz with the same magnitude and in constant proportion with the reference ultrasound-measured diameter (dashed line).

The simulation study resulted in impedance spectra for three different diameters within the frequency range of 1 kHz–2 MHz. These results explain why the diameter varies when estimated from impedance at different frequencies. To understand the difference in diameter estimated from measured impedance at different frequencies using (6), the ultrasound-measured diameters of subject 1 were used to interpolate the impedance spectra from the simulation (Figure 6a).

The morphology of each of the diameter waves follows that of the ultrasound-measured diameter. However, the mean magnitudes and the peak-to-trough amplitudes of the estimated diameters increase with frequency. This is due to the decreasing magnitudes of the impedance at higher frequencies and very small differences in the impedance spectra at frequencies around 2 MHz, as is clear from the simulation results. The trend of increasing diameter values with simulation frequency can also be seen in, and is symmetrically opposite to, that observed for the impedance spectra, as impedance is inversely proportional to artery diameter. However, the increasing trend is not so significant at lower frequencies (1–250 kHz). The comparison of these diameters with the reference ultrasound diameter has been more clearly represented in Figure 6b.

### 5.2. Comparing Ultrasound Measurements with Impedance Measurements

**Figure 7:**
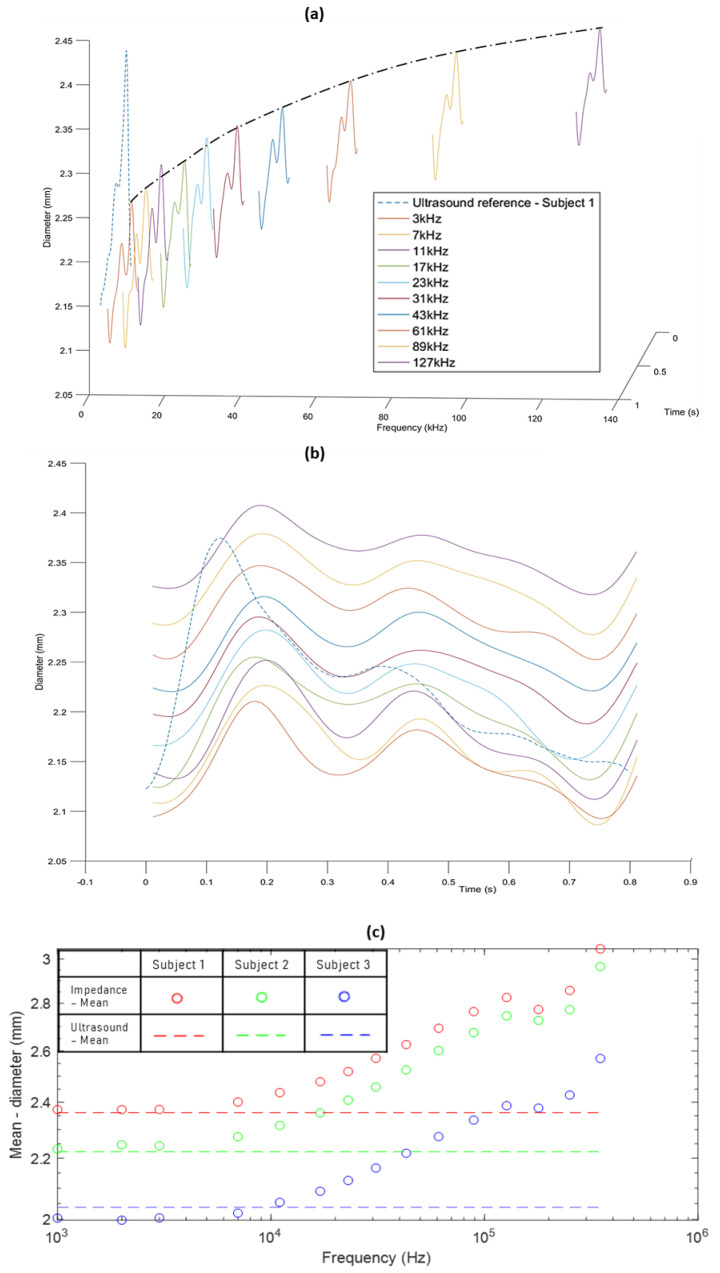

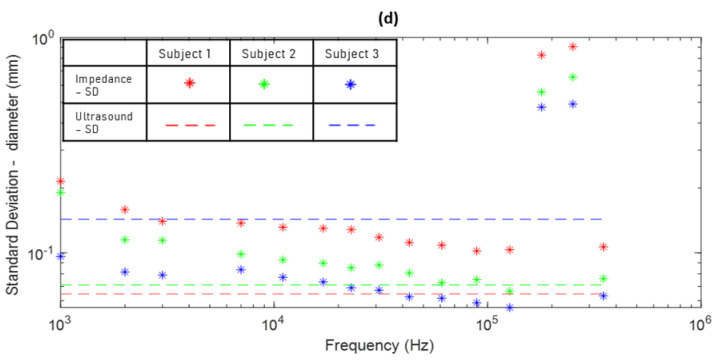
(**a**) Increasing trend of diameters obtained from Quadra^®^ impedance spectroscopy impedance measurements compared to the ultrasound-measured diameter for subject 3 (dashed line). (**b**) Comparison of the amplitudes of impedance-derived diameters with the ultrasound diameter for subject 3. (**c**) Comparison of mean of impedance-derived diameters with mean ultrasound diameter for all three subjects across all frequencies. (**d**) Comparison of standard deviation of impedance-derived diameters with standard deviation of ultrasound diameters for all three subjects across all frequencies.

Diameters calculated from the impedance measured using the Quadra^®^ impedance spectroscopy device were also compared to the ultrasound reference diameter, as can be seen in Figure 7a,b for subject 3. Similar behaviour was observed in the case of the other two subjects.

The diameters derived from the measured impedance show a similar increase in the trend of the diameter values as obtained by the simulation-derived diameters. However, the trend tends to converge at a frequency of 127 kHz in the latter case as compared to 2 MHz in the former case. This difference is possibly due to differences between the dispersion behaviour of the simulation study and the impedance measurements on human subjects. For the simulation and phantom studies, the three tissue layers—fat, muscle and the artery—were clearly separated domains, and the dielectric properties for the model and the phantom were defined to be isotropic. In real human forearm tissues, the tissue layers are all perfused to some extent with blood, and anisotropic tissue properties are expected.

The peak-to-trough amplitudes of the impedance-obtained diameters were significantly different from the ultrasound measurements. Along with considering average values for calculating *k* and *c* in (6), this can also be explained by differences between dielectric properties of the human forearm compared to the values used in the simulation. With regards to the morphology, impedance and ultrasound diameter waveforms are similar but show some apparent phase changes, as seen in Figure 7b. This may partly be due to the pulse wave and pulse rate changing between the time of the impedance and ultrasound measurements. However, some of the differences in shape are likely to indicate a need for further improvement in the impedance-derived diameters reflecting the pulsatile blood flow response. One focus should be on efficiently determining the constants *k* and *c*, which have been approximated for this initial investigation.

## 6. Conclusions

This study aimed to investigate the novel prospects of using EIS for estimating the changes in the left radial artery due to pulsatile blood flow in order to determine its feasibility of being employed in practice. The objective was to identify whether the simulation, phantom and analytical models, discussed here and previously, bear any resemblance to real life.

The results obtained indicate that the use of multiple frequency measurements may fundamentally aid in improving the accuracy of the otherwise conventionally employed SF-BIA approach adopted for hemodynamic monitoring. Here, we have compared the simulated impedance response at three artery diameter instances and the diameter calculated from the impedance measurements on three human subjects with the diameter measurements from the ultrasound. In the case of the simulation, the obtained diameter values at frequencies up to 250 kHz were found to be of the same order of magnitude and peak-to-trough amplitude as the ultrasound diameters for subject 1. Although diameters derived from impedance measurements differed in magnitude and peak amplitudes from the ultrasound reference, they offered the same trend and, hence, possibility of being applicable once improved.

Overall, the findings from this study indicate that not only is it possible to employ EIS for hemodynamic monitoring applications, it also offers good potential towards improvement of current systems. The results are comparable to those obtained from a high-resolution ultrasound measurement, which is promising. Eventually, this can lead towards accurately measuring arterial compliance, which directly affects blood pressure estimation. Future implications may be towards an alternative to ultrasound and a compact, cuffless blood pressure system. While these results lay a foundation for using MF-BIA in estimating diameter changes, the main challenge will be to effectively determine the relative contributions of the artery and surrounding tissue domains. It is obvious that this investigation must be followed by a larger clinical study involving different factors. Additionally, a consistent emphasis must be laid on improving the estimation of constants *k* and *c* for improvement of this methodology. Moreover, the impedance phase variations have not been studied within the scope of this investigation and may well be used to contribute towards an effective model to derive diameter from the impedance spectra. One of the considerations may also be towards a frequency selection or weighted frequency contribution criteria for calculating diameter from several impedance measurements at different frequencies.

## References

[1] PáVek K., Boska D., Selecký F.V., Sauberer A. (1964). Measurement of cardiac output by thermodilution with constant rate injection of indicator. Circ. Res..

[2] Fegler G. (1957). The reliability of the thermodilution method for determination of the cardiac output and the blood flow in central veins. Q. J. Exp. Physiol. Cogn. Med. Sci..

[3] Conway J., Lund-Johansen P. (1990). Thermodilution method for measuring cardiac output. Eur. Heart J..

[4] Opotowsky A.R., Hess E., Maron B.A., Brittain E.L., Barón A.E., Maddox T.M., Alshawabkeh L.I., Wertheim B.M., Xu M., Assad T.R. (2017). Thermodilution vs estimated fick cardiac output measurement in clinical practice: An analysis of mortality from the veterans affairs clinical assessment, reporting, and tracking (VA CART) program and vanderbilt university. JAMA Cardiol..

[5] Ihlen H., Amlie J.P., Dale J., Forfang K., Nitter-Hauge S., Otterstad J.E., Simonsen S., Myhre E. (1984). Determination of cardiac output by Doppler echocardiography. Heart.

[6] Darmon P.-L., Hillel Z., Mogtader A., Mindich B., Thys D. (1994). Cardiac output by transesophageal echocardiography using continuous-wave Doppler across the aortic valve. Anesthesiol. J. Am. Soc. Anesthesiol..

[7] Muhiudeen I.A., Kuecherer H.F., Lee E., Cahalan M.K., Schiller N.B. (1991). Intraoperative estimation of cardiac output by transesophageal pulsed Doppler echocardiography. Anesthesiol. J. Am. Soc. Anesthesiol..

[8] Giannotti G., Mondillo S., Galderisi M., Barbati R., Zaca V., Ballo P., Agricola E., Guerrini F. (2005). Hand-held echocardiography: Added value in clinical cardiological assessment. Cardiovasc. Ultrasound.

[9] Mjolstad O.C., Andersen G.N., Dalen H., Graven T., Skjetne K., Kleinau J.O., Haugen B.O. (2013). Feasibility and reliability of point-of-care pocket-size echocardiography performed by medical residents. Eur. Heart J. Cardiovasc. Imaging.

[10] Ramsingh D., Alexander B., Cannesson M. (2013). Clinical review: Does it matter which hemodynamic monitoring system is used?. Crit. Care.

[11] Van Lelyveld-Haas L.E.M., van Zanten A.R.H., Borm G.F., Tjan D.H.T. (2008). Clinical validation of the non-invasive cardiac output monitor USCOM-1A in critically ill patients. Eur. J. Anaesthesiol..

[12] Denniston J.C., Maher J.T., Reeves J.T., Cruz J.C., Cymerman A., Grover R.F. (1976). Measurement of cardiac output by electrical impedance at rest and during exercise. J. Appl. Physiol..

[13] Allen M.T., Fahrenberg J., Kelsey R.M., Lovallo W.R., Doornen L.J. (1990). Methodological guidelines for impedance cardiography. Psychophysiology.

[14] Cheney M., Isaacson D., Newell J.C. (1999). Electrical impedance tomography. SIAM Rev..

[15] Kotre C.J. (1997). Electrical impedance tomography. Br. J. Radiol..

[16] Bayford R.H. (2006). Bioimpedance tomography (electrical impedance tomography). Annu. Rev. Biomed. Eng..

[17] Kerner T.E., Paulsen K.D., Hartov A., Soho S.K., Poplack S.P. (2002). Electrical impedance spectroscopy of the breast: Clinical imaging results in 26 subjects. IEEE Trans. Med. Imaging.

[18] Mager J.R., Sibley S.D., Beckman T.R., Kellogg T.A., Earthman C.P. (2008). Multifrequency bioelectrical impedance analysis and bioimpedance spectroscopy for monitoring fluid and body cell mass changes after gastric bypass surgery. Clin. Nutr..

[19] Gotshall R.W., Wood V.C., Miles D.S. (1989). Comparison of two impedance cardiographic techniques for measuring cardiac output. Ann. Biomed. Eng..

[20] Muzi M., Jeutter D.C., Smith J.J. (1986). Computer-automated impedance-derived cardiac indexes. IEEE Trans. Biomed. Eng..

[21] Bloch K.E., Russi E.W. (1997). Comparison of impedance cardiography to invasive techniques for measurement of cardiac output. Am. J. Cardiol..

[22] Judy W.V., Powner D.J., Parr K., Demeter R., Bates C., Marshall S. (1985). Comparison of electrical impedance and thermal dilution measured cardiac output in the critical care setting. Crit. Care Med..

[23] Secher N.J., Thomsen A., Arnsbo P. (1977). Measurement of rapid changes in cardiac stroke volume. An evaluation of the impedance cardiography method. Acta Anaesthesiol. Scand..

[24] Miyamoto Y., Takahashi M., Tamura T., Nakamura T., Hiura T., Mikami M. (1981). Continuous determination of cardiac output during exercise by the use of impedance plethysmography. Med. Biol. Eng. Comput..

[25] Enghoff E., Lovheim O. (1979). A comparison between the transthoracic electrical impedance method and the direct Fick and the dye dilution methods for cardiac output measurements in man. Scand. J. Clin. Lab. Investig..

[26] Tang W.W. (2009). Impedance monitoring in heart failure: Are we really measuring hemodynamics?. Am. Heart J..

[27] Kubicek W.G. (1968). Minnesota Impedance Cardiograph Model 303.

[28] Kubicek W.G., Patterson R.P., Witsoe D.A. (1970). Impedance cardiography as a noninvasive method of monitoring cardiac function and other parameters of the cardiovascular system. Ann. N. Y. Acad. Sci..

[29] Mohapatra S.N. (1981). Non-Invasive Cardiovascular Monitoring by Electrical Impedance Technique.

[30] Mohapatra S.N. (1988). Impedance cardiography. Encycl. Med. Devices Instrum..

[31] Anand G., Lowe A., Al-Jumaily A.M. (2016). Simulation of impedance measurements at human forearm within 1 kHz to 2 MHz. J. Electr. Bioimpedance.

[32] Anand G., Lowe A., Al-Jumaily A. (2019). Tissue phantoms to mimic the dielectric properties of human forearm section for multi-frequency bioimpedance analysis at low frequencies. Mater. Sci. Eng. C Mater. Biol. Appl..

[33] Webster J.G. (2011). Medical Instrumentation Application and Design.

[34] Anand G., Lowe A., Al-Jumaily A.M. (2016). Parametric electrical modelling of human forearm simulation response using multi-frequency electrical bioimpedance. J. Biosens. Bioelectron..

